# Crack Assessment of Wheel Hubs via an Ultrasonic Transducer and Industrial Robot

**DOI:** 10.3390/s18124336

**Published:** 2018-12-08

**Authors:** Hanming Zhang, Chunguang Xu, Dingguo Xiao

**Affiliations:** School of Mechanical Engineering, Beijing Institute of Technology, Beijing 100081, China; xucg@bit.edu.cn (C.X.); xiaodg@bit.edu.cn (D.X.)

**Keywords:** robot-assisted, ultrasonic transducer, castings and forgings

## Abstract

Crack assessment when making fitness-for-service decisions requires a thorough examination of crack location and size in critical areas. An ultrasonic transducer is used for such assessments, but traditional methods cannot cope with complex rotators, such as wheel hubs. We present a model of robot-assisted crack growth assessment in wheel hubs. We integrate a six-degrees-of-freedom (DOF) industrial robot and a turntable to form a robot-assisted ultrasonic testing (UT) system that does not use traditional UT equipment. Ultrasonic beams are focused at certain depths appropriate for achieving maximum sensitivity. We quantitatively analysed wheel hubs with longitudinal and transverse series of pre-cracks, and concluded that our system autonomously detected cracks.

## 1. Introduction

Casting is commonly used to produce wheel hubs. This involves metal smelting, pouring, and freezing. The performance of cast products is influenced by pressure, temperature, geometry, and chemistry [1]. Wheel hubs bear running dynamic, impact, cyclic, and random loads, all of which affect service life. Hübner et al. explored the fracture behaviour of wind turbine components under cyclic and random loads [2], concluding that load history was key for lifespan prediction. X-rays can detect product defects. Principe et al. compared paired samples to ultrasonic testing (UT) and X-ray testing, where ultrasound revealed small defects misinterpreted by X-rays [3].

UT facilitates material characterisation and defect detection [4]. Many UT applications have been reported. For example, the ultrasonic characteristics of cast irons with different nodularities and matrix structures were reported by Collins et al. [5]. Similarly, it was found that UT could be used to characterize the percent of vermicular graphite [6]. Besides, ultrasound is commonly used for defect detection, affording good sensitivity and penetration. However, some disadvantages are apparent. For example, the incident angle is difficult to control on a complex surface and echoic waves are easily disturbed by noise, causing cracks to be missed or falsely detected [7]. In addition, a stand block must be used as an evaluation reference.

Traditionally, objects were scanned manually using an ultrasonic transducer, and data were thus affected by human factors. Automatic scanning methods were later developed, such that the ultrasonic transducer was integrated with automatic production. X−Y−Z immersion C-scan systems are the most commonly used [8]. The object under test is fixed in a water tank in which a triaxial manipulator is installed (on the top), and which moves in three orthogonal directions. An ultrasonic transducer installed on the end of the manipulator scans the object to detect defects. Although this bridge design can follow contours via interpolation, the object must exhibit a simple and continuous geometry.

To improve scanning flexibility, robots have recently been introduced. Various movable and stationary robotic platforms resolve the deficiencies of existing mechanisms. For example, Summan et al. designed a miniature robotic vehicle equipped with an air-coupling transducer to test large curved surfaces, but the vehicle is not commonly used [9]. In contrast, general six-degrees-of-freedom (DOF) industrial robots are now available off-the-shelf, and are widely used. Several key problems encountered when integrating robots with ultrasonic transducers, including tool path planning [10], phased-array C-scanning [11], and multi-robot collaboration [12], have been addressed by Mineo et al.

We developed a robot-assisted UT system assessing cracks in wheel hubs. The design is modular in nature, facilitating incorporation into high-throughput assembly lines. A turntable allows the system to efficiently test rotators. System effectiveness is verified using specimens containing a series of pre-cracks.

## 2. Experimental Method and Configuration

### 2.1. Analysis of Cracks in Wheel Hubs

**Figure 1:**
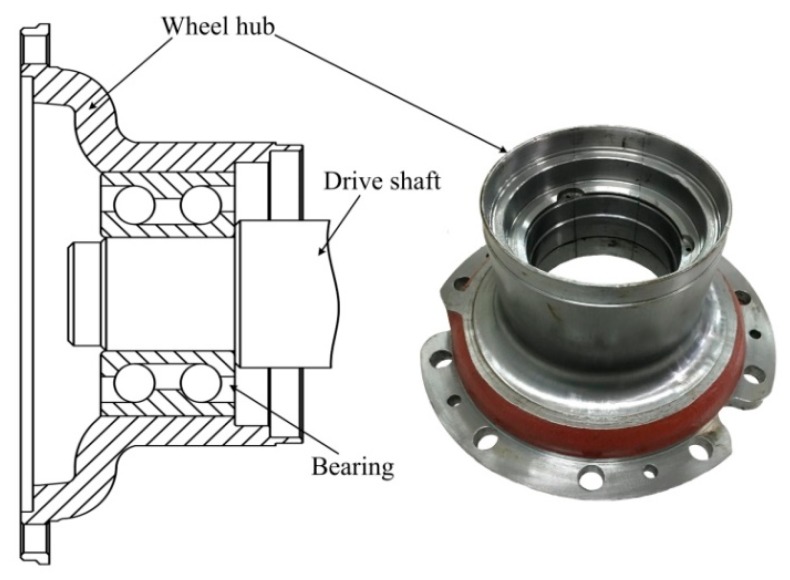
Assembly of a wheel hub with other objects.

The wheel hubs assessed herein were those of a heavy-duty train (Figure 1). The hubs connect the drive shaft to the wheels, and thus bear simultaneous axial and radial loads. Initially, cracks principally appear on matching surfaces and areas of stress concentration after long-term use. In the absence of regular assessment, the cracks grow continuously and finally cause hub fracture.

The principal causes of crack growth or fracture are:

- Heat damage. Friction-heat is generated within bearings during high-speed operation and is then transmitted to the hub. The area most affected is the interface between the hub and the outer bearing ring. There are two causes of heat cracking: a quenching effect caused by the rapid cooling of a hot surface, and thermal stress caused by the uneven heating of a local surface.
- Fatigue damage. Micro-cracks appear in wheel hubs under long-term cyclic loads, but do not affect normal operation until they become enlarged. Fatigue cracks are generally sub-surface in location and extend both inwards and outwards, finally causing accidents. Crack growth is remarkably random in nature, rendering crack detection difficult. Complete scans are ideally required.
- Impact damage. Although wheel hubs feature many micro-cracks, these do not expand rapidly. However, the impact load accelerates crack growth, rapidly reducing effectiveness. As impact loads will greatly reduce service life, such loads should be avoided.

According to linear elastic fracture mechanics, the mechanism of crack growth is generally considered to be relative to the external stress and crack size. Irwin first used a stress intensity factor, *K_I_*, to estimate the intensity of the stress field at the crack tips [13]. When *K_I_* exceeded a critical value, the crack tip exhibited unstable growth, and thus a brittle fracture occurred. The critical parameter characterising the resistance of a material to unstable growth is the fracture toughness, *K_mat_*. That means when *K_I_* *>* *K_mat_*, the crack will turn to unstable. Certain national testing standards (e.g., BS 7448-4:1997 [14], BS 7910:2013 [15]) specify methods for estimation of *K_mat_*. A simplified equation linking the fracture toughness and the crack size is:

(1) $$a_{\mathrm{pl}}=\frac{1}{\pi }{\left(\frac{K_{mat}}{\sigma_{Y}}\right)}^{2}$$

where *σ_Y_* is the yield strength of the material and *a_pl_* is the critical value of the crack size. Equation (1) is derived from British Standard (BS) 7910:2013, and this standard specifies methods used to assess fracture resistance based on defect detection.

It is concluded that cracks are the principal defects causing wheel hub failure after long-term operation. The crack size is negatively related to fracture-resistance. If the crack size exceeds the critical value *a_pl_*, the wheel hub is not fit for service.

### 2.2. Crack Test Methodology

UT is commonly used to detect layered defects, the reflective surfaces of which lie parallel to the incident surface. Echoes from such defects are strong and easily identified. However, cracks caused by cyclic loading and stress concentration grow vertically to the incident surface, rendering it difficult to identify echoes (some of which may be missed). We used a focused shear wave (converted from a longitudinal wave) to detect wheel hub cracks. The cracks are divided into transverse and longitudinal types. A different approach was used to detect each type of crack. Both the incident angle and the water path were considered when focusing the shear wave at different depths, improving echo identification.

#### 2.2.1. Transverse Crack Test

**Figure 2:**
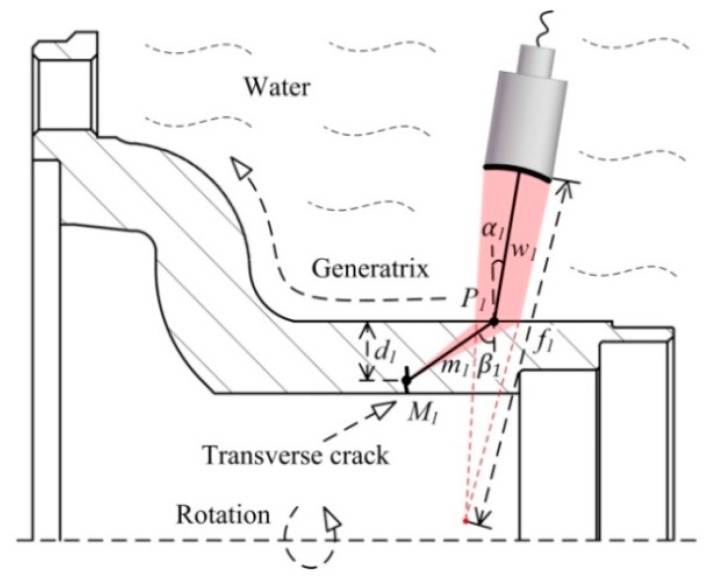
Transverse crack testing of a wheel hub.

The method used to test for transverse cracks is shown in Figure 2. The focusing transducer and wheel hub are immersed in water. The focal length of the transducer in water is *f_1_*. A longitudinal wave is emitted by the transducer to enter the material at incident angle *α_1_*_,_ at point *P_1_*. Hence the angle of incidence is oblique. *α_1_* lies between the first and second critical angles:

(2) $$\frac{c_{w}}{c_{ml}}<\sin\alpha_{1}<\frac{c_{w}}{c_{ms}}$$

where *c_w_* is the wave velocity in water, and *c_ml_* and *c_ms_* are the longitudinal and shear wave velocities in the material, respectively (although only the shear wave is converted). The shear wave refraction angle is *β_1_*, in line with Snell’s Law:

(3) $$\sin\beta_{1}=\sin\alpha_{1}\times \frac{c_{ms}}{c_{w}}$$

The refracted beam is focused at point *M_1_*. The path of the beam in water is given by *w_1_* and that in the material by *m_1_*. The relationship between *w_1_* and *m_1_* is:

(4) $$\frac{f_{1}-w_{1}}{m_{1}}=\frac{c_{ms}}{c_{w}}$$

in line with the proportional relationships of sound velocities in different materials. The depth of *M_1_* from the incident surface is *d_1_*:

(5) $$d_{1}=m_{1}\cos\beta_{\;}{}_{1}=\frac{c_{w}}{c_{ms}}\left(f_{1}-w_{1}\right)\cos\beta_{\;}{}_{1}$$

as inferred using the triangle relationship and Equation (4), the beam can be focused at different depths by adjusting *w_1_*. However, *α_1_* must satisfy Equation (2) during adjustment, ensuring that only the shear wave is converted within the material.

The ultrasonic beam can be focused at a specific depth using the method shown in Figure 2. Sound energy near the focus is higher than in other areas. If the focus is positioned on the cracks, the amplitude of the echo wave will get bigger and the sensitivity of echo identification will be improved. The wheel hub revolves around an axis, and the transducer moves along the generatrix at a fixed incidence angle *α_1_*. These two motions combine to create a screw scan detecting transverse cracks.

#### 2.2.2. Longitudinal Crack Test

**Figure 3:**
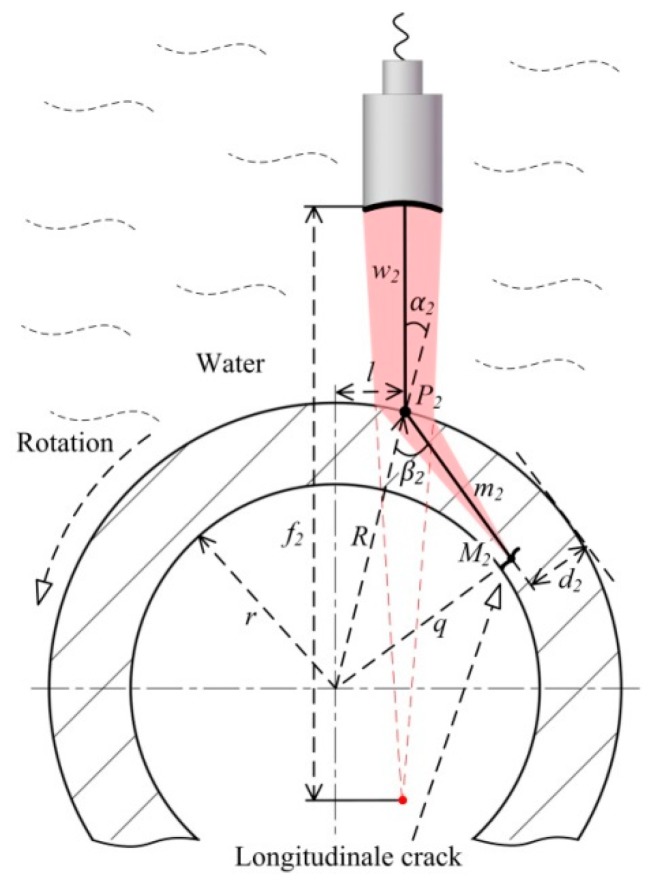
Longitudinal cracking test for a wheel hub.

The method used to explore longitudinal cracking is shown in Figure 3. The means by which an oblique incidence is attained differs from that of the transverse crack test. The focusing transducer is vertically immersed in water. The offset *l* between the transducer axis and that of the wheel hub ensures that the longitudinal wave enters the material at point *P_2_* at an incident angle *α_2_*. A triangular relationship is evident between *l* and *α*_2_:

(6) $$\sin\alpha_{2}=l/R$$

where *R* is the external radius of the wheel hub. *α*_2_ is set between the first and second critical angle by adjusting *l* over a certain range, converting only the shear wave within the material. The shear wave is focused at point *M_2_*. The distance between *M_2_* and the wheel hub axis is *q*. *w_2_* and *m_2_* reflect the water and material paths, respectively. Their relationship is similar to that of Equation (4):

(7) $$\frac{f_{2}-w_{2}}{m_{2}}=\frac{c_{ms}}{c_{w}}$$

The *M_2_* depth *d_2_* from the incident surface is:

(8) $$d_{2}=R-q$$

*q* can be calculated using the cosine law:

(9) $$q^{2}=R^{2}+m_{2}^{2}-2Rm_{2}\cos\beta_{2}=R^{2}+{\left[\left(f_{2}-w_{2}\right)\frac{c_{w}}{c_{ms}}\right]}^{2}-2R\left[\left(f_{2}-w_{2}\right)\frac{c_{w}}{c_{ms}}\right]\cos\beta_{2}$$

Thus, the beam can be focused at different depths by adjusting *w_2_*. *l* must be maintained within a certain range during such an adjustment. In Figure 3, the wheel hub revolves around the axis, and the transducer moves along the generatrix axis in the presence of a normal offset. These two motions combine to create screw scans detecting longitudinal cracks.

### 2.3. Configuration of the Test System

**Figure 4:**
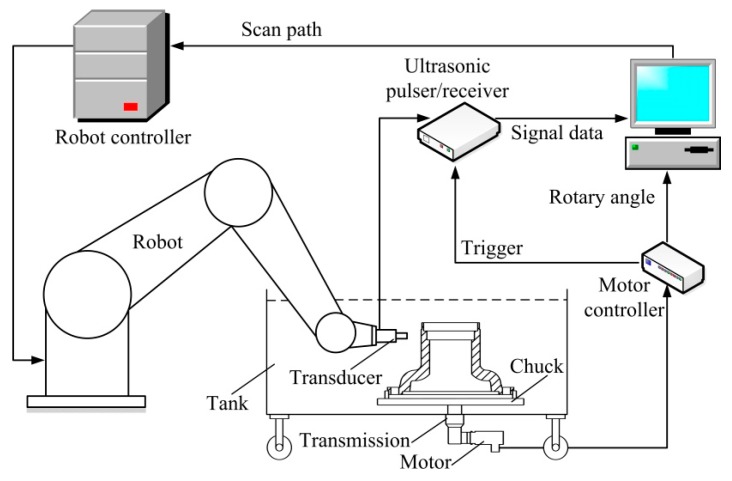
Configuration of the robot-assisted ultrasonic testing (UT) system.

The six-DOF industrial robots were flexible, ensuring that the incidence angle on a complex curved surface was maintained more effectively than by traditional mechanisms. The configuration of the system is shown in Figure 4. It is divided into the mechanism, UT, and motion control modules. A computer generates a scan path offline and combines signal data with the space coordinates of the incidence points, forming an RGB pseudo-colour map termed a C-scan image.

#### 2.3.1. Mechanism

The use of six-DOF articulated robots greatly aids scanning of curved surfaces [16]. Although the robots are flexible, they may not reach all points on curved surfaces. According to robotic kinematics, there are several singular and interference points that robots cannot reach [17]. Our test object was a revolving body with a curved surface. The robot can easily be impeded at these points when scanning around a wheel hub.

To solve this problem, we integrated the robot with an independent turntable (Figure 4). The robot was fixed to the ground. The turntable featured a chuck, a transmission, a motor, and a tank, and was moveable. The tank had four wheels for positional adjustment. The motor was under the bottom of the tank and was connected to the main shaft of the transmission via a watertight bearing. The chuck ensured that the wheel hub was centred over the turntable axis. It was essential to correctly position the turntable gyration centre and the robot-base-frame to ensure accurate testing. A laser-tracker, industrial charge-coupled device (CCD) camera, or ball-bar can be used for positional calibration.

#### 2.3.2. Ultrasonic Testing

Earlier UT equipment featured an ultrasonic pulser/receiver, a transducer, and an oscilloscope. Defects were manually identified by reference to echo signals on the oscilloscope and a stand block. Automatic testing replaced the oscilloscope with a computer and an A/D converter inserted into the peripheral component interconnect (PCI) computer slot, which digitalises echo signals. The converter featured a gating circuit and onboard buffer memory. Characteristic gate peaks and times-of-flight were captured by the gating circuit. These values, and the digitalised signal data, were sent to the computer memory via the onboard buffer memory. A gating circuit with onboard buffer memory is essential for high-speed scanning. The pulser/receiver was externally triggered by the I/O port on the motor controller, matching the echo signal data with the corresponding rotary angle. One-to-one correspondence is essential for useful post-processing in terms of tomography, offline review, and data analysis.

#### 2.3.3. Motion Control

The motion control module included the robot and motor controllers. The robot controller was connected via the Ethernet to the computer, allowing scan path transmission. The motor controller was connected to the computer via a CAN Open Bus, facilitating real-time rotary angle transmission. Data synchronisation was achieved by triggering the pulser/receiver at certain rotational steps. The scanning velocity was limited principally by the maximum trigger frequency of the pulser/receiver.

## 3. Experiment Results and Discussion

**Figure 5:**
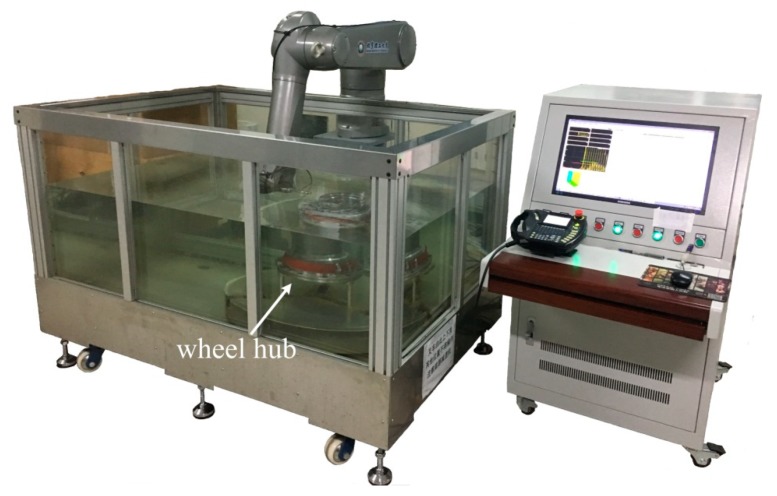
Overview of the robot-assisted UT system.

The system in Figure 5 was created to evaluate the wheel hub in Figure 1 immersed in water. The hub was made of grade C60E steel (conforming to the ISO standard). A single-element ultrasonic transducer (Panametrics-NDT) was installed on the end-effector of a Staubli industrial robot and used to scan the object. Detailed information of the components was listed in Table 1. The longitudinal and shear wave velocities of the steel were *c_ml _= *5860 m/s and *c_ms _= *3230 m/s, respectively. Only the shear wave was converted when the incidence angle was 15–27°, as calculated by Equation (2).

**Figure 6:**
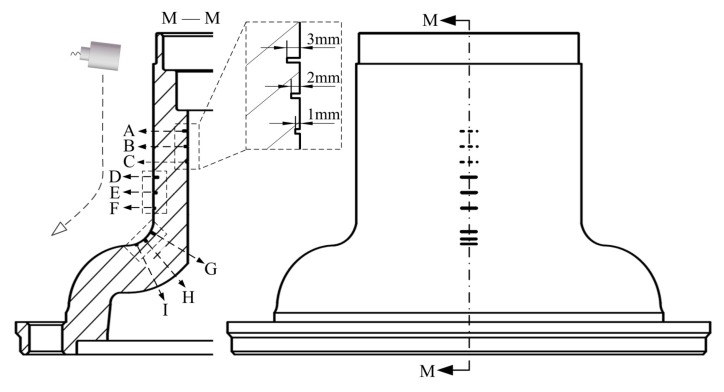
The distribution of transverse cracks.

Two types of wheel hub pre-cracks were machined to explore the performance of the system. One wheel hub featured the nine transverse pre-cracks labelled A to I in Figure 6. The cracks were divided into three groups, in the inner wall, the outer wall, and a corner along the generatrix circled in the sectional view M–M. Each group contained cracks of three different depths: 1, 2, and 3 mm.

**Figure 7:**
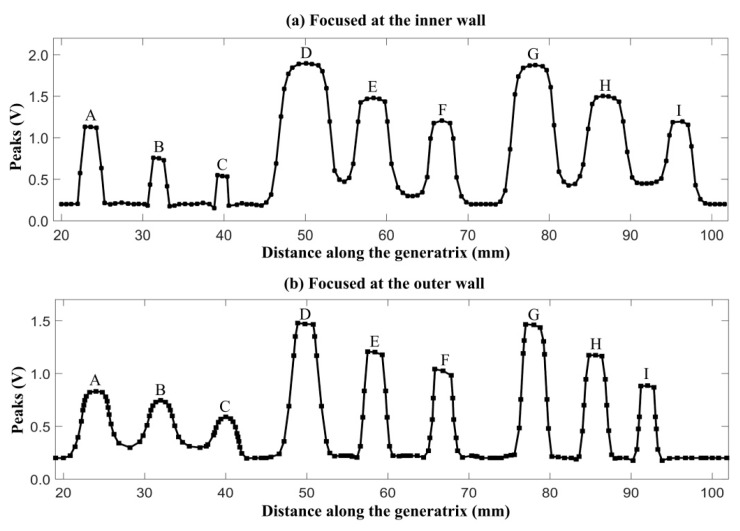
Echo peaks acquired using different water paths (8.82 and 91.29 mm).

In Figure 6, the transducer moved along the generatrix and traversed the nine cracks. The ultrasonic pulser/receiver was triggered by an internal clock. The echo peaks reflected by the cracks are plotted in Figure 7. The two curves were acquired using different water paths. The nine crests of each curve (labelled A to I) correspond to the cracks labelled in Figure 6.

Section 2.2 showed that the depth of focus was determined by the water path at a fixed incident angle. In Figure 6, the incident angle was held at 20°. The beam was focused at two depths (on the inner and outer wall), denoted *d_1a_*= 27.0 mm and *d_1b_*= 3.0 mm, respectively. The corresponding water paths were calculated using Equation (5) and were* w_1a_*= 8.82 mm and *w_1b_*= 91.29 mm, respectively. In Figure 7a, the beam was focused at the inner wall. The crests labelled A to C were sharper than those labelled D to I. In Figure 7b, the beam was focused on the outer wall and sharpness of the crests was reversed. Thus, the echoes reflected by cracks near the focus were more sensitive.

**Figure 8:**
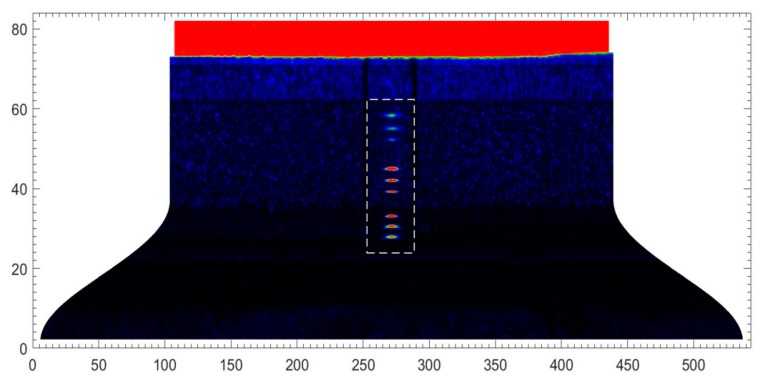
C-scan image of transverse cracks developing when the water path was 91.29 mm.

**Figure 9:**
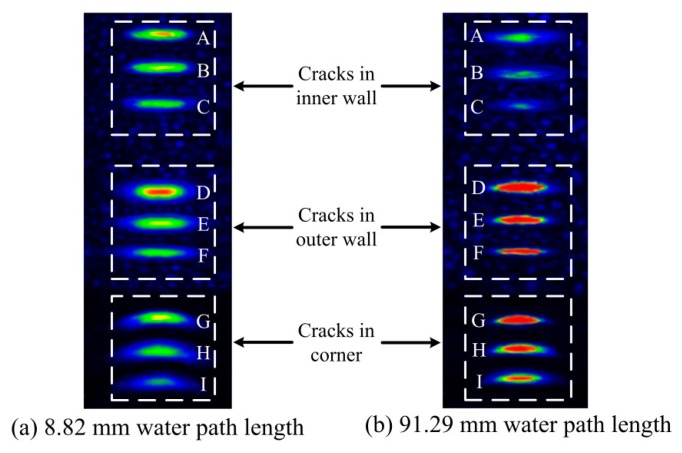
Comparison of transverse cracks acquired using two water paths.

The wheel hub in Figure 6 was fully scanned. The ultrasonic pulser/receiver was externally triggered by the turntable motor controller. The test result using a 91.29 mm water path is shown in Figure 8. This C-scan image contains both positional and peak information. Section 2.2 showed that the screw−scan is used to cover the tested object. Hence, the C-scan image is an expanded view, where the abscissa represents the circumference, and the ordinate height. Each pixel was derived from a single trigger. Pixel RGB values were determined by the peaks of the echo signals. Image resolution was determined by robot motional velocity and turntable rotational velocity. Nine transverse pre-cracks were circled. Partial enlargements of the circled area acquired using water paths of 8.82 mm and 91.29 mm are shown in Figure 9 for comparison.

Letters A to I in Figure 9 correspond to the cracks labelled in Figure 6. The beam was focused on the inner wall using 8.82 mm water path length. Hence, cracks A to C in Figure 9a are clearer than in Figure 9b. Then, the beam was focused on the outer wall using 91.29 mm water path length. Hence, cracks D to I in Figure 9b are clearer than in Figure 9a. Thus, the result image better reflects real size when the beam is focused near cracks.

**Figure 10:**
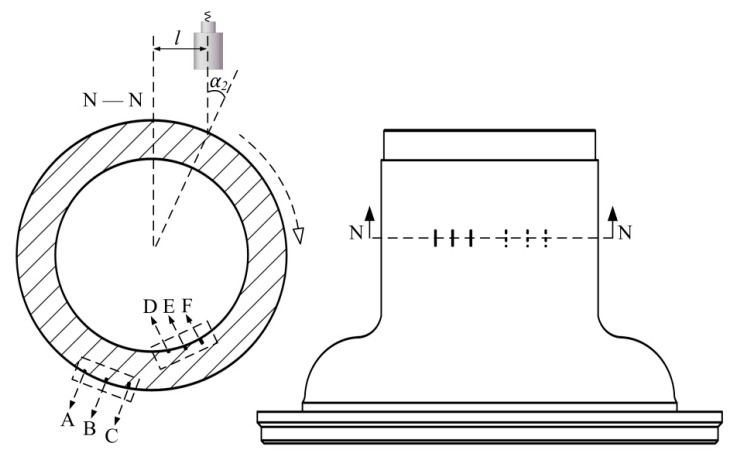
Distribution of longitudinal cracks.

We also scanned a wheel hub containing two groups of longitudinal pre-cracks (Figure 10). The crack depths were the same as those of Figure 6 (1, 2, and 3 mm). The transducer remained at a distance *l* from the axis and the hub revolved around the axis, as shown in sectional view N–N.

**Figure 11:**
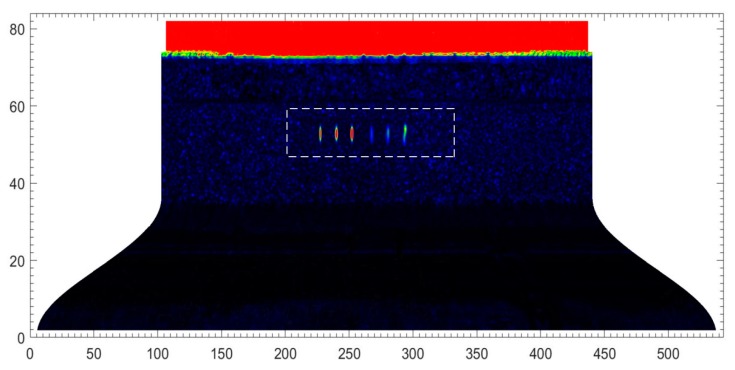
A C-scan image of longitudinal cracks when the water path was 21.27 mm in length.

When testing for longitudinal cracks, the required oblique incidence was created by applying a normal offset. In Figure 10, *l* is adjusted in line with Equation (6), with the incident angle held at 20°. A wheel hub containing longitudinal cracks (Figure 10) was fully scanned. The beam was focused on the inner wall (*d_2_*= 27.0 mm). The corresponding water path length* w_2_*was 21.27 mm, calculated using Equations (8) and (9). The test result is shown in Figure 11, longitudinal cracks were effectively detected.

## 4. Conclusions

We developed a new, robot-assisted UT system for detecting cracks in curved rotators, such as wheel hubs. A within-tank turntable was used to expand the robot workspace. Heat, fatigue, and impact were the principal causes of crack growth (both transverse and longitudinal). Each type of crack was detected differently. The focus depth of the ultrasonic beam was analysed to optimise the sensitivity of the echo wave. The focus depths were determined by the water paths and certain geometric relationships were in play. We demonstrated the effectiveness of our system and the relevant geometric relationships. The system was remarkably flexible in terms of fixed incidence angle maintenance. Cracks evident in C-scan images were particularly noticeable near the foci, reflecting real crack sizes. Cracks with more than 1 mm in width and less than 29 mm in depth were able to be detected using this system. Our future work will be aimed at calibrations that optimise the relative positioning of the turntable gyration centre and the robot base frame.

## Figures and Tables

**Table 1:** Main components of the robot-assisted UT system.

Apparatus	Brand	Model	Property
Robot	Staubli (Pfäffikon, Switzerland)	TX90XL-HE	Repeatability 0.035 mm
Motor of the turntable	Parker (Cleveland, OH, USA)	SME Series	Power 1.57 kW, Torque 6 Nm
Motor controller	Parker (Cleveland, OH, USA)	Complex 3 Series	Control via CAN Open
Ultrasonic pulser/reciver	Olympus (Tokyo, Japan)	5077PR	Receiving bandwidth 1 kHz–35 MHz
Ultrasonic transducer	Olympus (Tokyo, Japan)	V319-SU-F4.0IN	Centre frequency 15 MHz
A/D converter	Acquisition Logic (Chantilly, VA, USA)	AL12200	Sampling rate 100 MHz
